# *In vitro* inhibition of *Streptococcus mutans* by cardamom essential oil

**DOI:** 10.3389/froh.2026.1796712

**Published:** 2026-05-11

**Authors:** Nanki J. Singh, Gizem Kezer, Marcus A. Horn, Silke Hillebrand, Tuba Esatbeyoglu

**Affiliations:** 1 Department of Molecular Food Chemistry and Food Development, Institute of Food and One Health, Gottfried Wilhelm Leibniz University Hannover, Hannover, Germany; 2 Institute of Microbiology, Gottfried Wilhelm Leibniz University Hannover, Hannover, Germany; 3 Symrise AG, Holzminden, Germany

**Keywords:** anticariogenic activity, biofilm inhibition, dental caries, *Elettaria cardamomum*, spice, virulence gene expression

## Abstract

Dental caries is a biofilm-related disease that disproportionately affects low-income populations due to limited access to expensive treatments. Natural products offer a promising and affordable alternative for preventing cavities. This study investigated the antibacterial and antibiofilm activity of cardamom essential oil (CEO), which contains bioactive compounds such as 1,8-cineole, linalool, and sabinene, against *Streptococcus mutans*, the primary cariogenic bacterium. Antimicrobial activity was evaluated using agar well diffusion, broth microdilution, biofilm (crystal violet), and cell viability (MTT) assays. The effects of CEO on six virulence-associated genes (*gtfB, gtfC, gtfD, ldh, gbpB,* and *vicR*) were analyzed using quantitative PCR. Minimum inhibitory and bactericidal concentrations of CEO were 2.5% and 5.0% (*v/v*), respectively. At 5.0%, biofilm formation and cell viability decreased by more than 75%, while virulence gene expression was upregulated, indicating a stress response. Among twelve tested essential oils, CEO showed the strongest inhibition of *S. mutans* growth and biofilm formation. These findings demonstrate the *in vitro* inhibitory potential of CEO against *S. mutans* and highlight its promise as a natural, accessible compound for future development of cost-effective oral care formulations. Further validation in polymicrobial and *in vivo* models will help to confirm and extend these findings toward potential anticariogenic applications.

## 1 Introduction

Dental caries is a disease that manifests itself through cavities in the teeth (1, 2). The cost of treatment is a burden not only on individuals but also on entire healthcare systems (3, 4). People in developing countries or people with a low socioeconomic status are particularly often affected due to insufficient access to prophylaxis (1, 2). Caries is caused by the interaction of various bacteria with the teeth. *Streptococcus mutans* (*S. mutans*) plays a major role (3, 5–10). It has adherence mechanisms for adhesion in conditions with and without sucrose, whereby the presence of sucrose leads to facilitated adhesion, and it enables the formation of a stable biofilm that can harbor other cariogenic bacteria (7, 8, 11, 12). This biofilm is composed of extracellular polymeric substances (EPS), which are primarily made of glucans synthesized by glucosyltransferases (encoded by *gtfB*, *gtfC*, and *gtfD*) from sucrose (13). These glucans contribute to the structural stability of the biofilm, which protects the bacteria from environmental stress and allows for the maintenance of suboxic or anoxic conditions (14). Under these conditions, *S. mutans* metabolizes carbohydrates via glycolysis to pyruvate, which is then fermented to acids such as lactic acid, acetic acid, and formic acid. These acids attack and dissolve tooth minerals, leading to caries (2, 6, 11, 12).

The formation of biofilms and acid production is closely linked to the expression of various virulence genes in *S. mutans*, including *gtfB, gtfC,* and *gtfD* (encodes glucosyltransferases responsible for soluble and insoluble glucan synthesis), *ldh* (encoding lactate dehydrogenase, which produces lactic acid), *gbpB* (encoding glucan-binding protein B, involved in biofilm integrity), and *vicR* (a regulator of virulence gene expression) (5). Established methods of caries prevention include the natural remineralization of teeth using minerals contained in saliva or fluoride from toothpaste, for example (3, 6, 12). The most common method of removing biofilms from tooth surfaces and preventing the production of acids is mechanical removal by tooth brushing, but antibacterial agents such as chlorhexidine are usually required if the bacterial load is too high (3, 6, 15, 16). However, its use is associated with numerous side effects, which discourages many patients from using it (16–19). Natural compounds such as plant extracts and essential oils are increasingly being investigated as alternative antimicrobial agents against *S. mutans* due to their low toxicity potential and wide availability (20, 21). However, the efficacy of these compounds may vary depending on factors such as variability in chemical composition, stability issues, and bioavailability (22). Furthermore, the fact that many natural compounds exhibit limited efficacy against bacteria within biofilms poses a significant limitation in the management of oral infections (13). This situation requires the investigation of more consistent and effective natural agents. In this regard, cardamom essential oil (CEO) emerges as a potential candidate.

Cardamom, also known as *Elettaria cardamomum* L. Matón var. *cardamomum*, is part of the Zingiberaceae family and is native to India, Sri Lanka, Guatemala and Tanzania (23–25). The plant has pods filled with oil-containing seeds (26). These seeds are used for cooking, but also in traditional medicine to treat e.g., abdominal pain, sore throats and oral diseases (23–25, 27). Indeed, it is common to chew cardamom pods, which releases the essential oil (EO), stimulates the salivary flow and increases the pH value in the mouth (23, 28). These effects are based on the bioactive components of the EO, which include 1,8-cineole, *α*-terpineol, linalool, linalyl acetate, D-limonene and sabinene (Supplementary Figure S1) (24, 27). However, the question remains whether cardamom essential oil (CEO) is the most effective EO in terms of anticariogenic properties compared to other EOs.

In this study, the effects of 12 essential oils derived from various foods on *S. mutans*, the primary causative agent of dental caries, were evaluated for their antibacterial activity through preliminary screening. Based on the findings, among these 12 essential oils, cardamom essential oil (CEO) was of particular interest due to its bioactive compounds associated with antimicrobial properties. However, unlike well-studied oils such as clove, cinnamon, and tea tree, CEO has received limited scientific attention, and its effects on *S. mutans* growth, biofilm formation, and virulence gene expression remain largely unexplored. Therefore, CEO was selected for further analysis, and this study aimed to comprehensively assess the inhibitory effects of CEO on *S. mutans in vitro*. Specifically, this study investigated its impact on bacterial growth, biofilm formation, and the expression of six virulence-associated genes (*gtfB, gtfC, gtfD, ldh, gbpB*, and *vicR*). The null hypothesis (H_0_) of this study is that cardamom essential oil has no significant effect on *S. mutans* growth and virulence gene expression, and does not differ significantly from the other essential oils tested. However, considering the known antibacterial properties of its main components, it is thought that CEO may inhibit *S. mutans* growth and modulate virulence gene expression, supporting its potential as a natural anticariogenic agent.

## 2 Materials and methods

### 2.1 Materials

#### 2.1.1 Samples

Twelve essential oils, cardamom (*Elettaria cardamomum*), ginger (*Zingiber officinale*), lemon (*Citrus limon*), lemon-scented gum (*Eucalyptus citriodora*), mandarin (*Citrus reticulata*), nutmeg (*Myristica fragrans*), peppermint (*Mentha piperita*)**,** southern blue gum (*Eucalyptus globulus*), spearmint (*Mentha spicata*), star anise (*Illicium verum*), tea tree (*Melaleuca alternifolia*), and wild mint (*Mentha arvensis*), were obtained from Symrise AG (Holzminden, Germany).

#### 2.1.2 Chemicals

Purified water was prepared using the PURELAB flex 3 (Veolia Water Technologies, Celle, Germany). Dimethyl sulphoxide, ≥99.8%, p.a., guanidine thiocyanate, ≥99% and sodium chloride were acquired from Carl Roth (Karlsruhe, Germany). Ammonium thiocyanate was obtained from Honeywell International Inc. (Morristown, NJ, USA), and nuclease-free water from Life Technologies Corp. (Austin, TX, USA). Phenol, water-saturated and stabilised, and TAE buffer 50X were ordered from ITW Reagents (Barcelona, Spain). Sodium acetate, anhydrous for analysis, brain heart broth, crystal violet and methylthiazolyldiphenyl-tetrazolium bromide were supplied by Merck (Darmstadt, Germany). Chloroform molecular biology reagent was provided by MP Biomedicals, INC. (Eschwege, Germany), and glycerin, ≥97% by VWR (Darmstadt, Germany). Ethanol, 99% denatured with 1% methyl ethyl, ketone and isopropanol were ordered from WALTER CMP (Kiel, Germany). M-MLV reverse transcriptase and M-MLV RT 5X Buffer were obtained from Promega (Walldorf, Germany). DNase I, RNase-free, 1 U/μL, 10X reaction buffer with MgCl_2_ for DNase I, dNTP set 100 mM solutions, PowerTrack SYBR green mastermix and SYBR Safe DNA gel stain were acquired from Thermo Fisher Scientific (Schwerte, Germany).

#### 2.1.3 Microorganisms

*Streptococcus mutans* (*S. mutans*) ATCC 25175 (DSMZ, Germany) was used as the test strain. Preliminary optimization showed that *S. mutans* grew equally well under standard incubator conditions without additional CO₂ supply compared to growth under 5% CO₂. For this reason, all cultures were incubated at 37°C in a standard incubator. For agar-based experiments, tryptic soy broth (TSB), commonly recommended for the cultivation of *S. mutans*, was used. For biofilm and gene expression analyses, brain heart infusion (BHI) supplemented with sucrose was selected because it supports stronger bacterial growth, mimics sugar-rich oral environments, and enables better comparability with previous studies.

#### 2.1.4 Serial dilution of *S. mutans*

Eight test tubes, one of which was filled with 3 g of glass beads (2.85–3.45 mm), were covered with Kapsenberg caps and sterilized by autoclaving in the VX-65 autoclave by Systec (Osnabrück, Germany). The test tube containing glass beads was filled with 10 mL of medium, and 9 mL of the identical medium were added to all remaining tubes. A sterilised inoculation loop was used to pick up colonies of the second subculture of *S. mutans*, and stirred in the glass bead-containing test tube to release cell material, which was then vortexed for 3 min. The optical density (OD) measured at 600 nm of the bacterial suspension in this tube was adjusted to values between 0.25 and 0.3 to ensure that the number of cells was approximately 1.0 × 10^8^ CFU/mL. OD-CFU relationships were determined in preliminary experiments. One milliliter was taken from the sample and transferred to another test tube with 9 mL of medium. This step was repeated until a dilution of 10^−6^ was reached. To determine the CFU/mL, 100 μL of the 10^−6^ and the 10^−5^ dilutions were pipetted onto two TSB agar plates respectively and spread evenly with Drigalski cell spreaders. After drying for 30 min, the petri dishes were incubated upside down for 2 d at 37°C.

### 2.2 Methods

A total of 12 essential oils were initially screened using the agar well diffusion method. Based on the results of this preliminary screening, CEO was selected for further analysis, and all following experiments were conducted using only CEO.

#### 2.2.1 Agar well diffusion assay for the evaluation of antibacterial activity

TSB agar [15 g/L casein peptone (pancreas hydrolysate), 5 g/L soy peptone (papain hydrolysate), 5 g/L NaCl, 15 g/L agar-agar] plates with a depth of ca. 6 mm were inoculated with 100 μL of the 10^−2^ dilution using Drigalski cell spreaders. The petri dishes were left to dry for 10 min. A pair of forceps, sterilised by flaming, was used to grip a sterile 1,000 μL pipette tip. The wider opening of the pipette tip (0.9 cm diameter) was pressed into the TSB agar three times. The holes were emptied with a sterile 1,000 μL pipette tip and sterile forceps before they were filled with 80 μL of sample. The samples were prepared by 20-fold and 40-fold diluting essential oils in ≥99.8% p.a. dimethyl sulphoxide, thus obtaining concentrations of 5.00% (*v/v*) and 2.50% *(v/v*)*.* A solvent control with 80 μL DMSO and a positive control with 80 μL of 1 μg/mL penicillin diluted in purified water were included. The pH values of the samples were tested using universal indicator paper 1–14 (Ahlstrom Germany, Baerenstein, Germany). The petri dishes were sealed with parafilm and left in the biological safety cabinet for 10 min. After being incubated at 37°C for 48 h, the diameters of the inhibition zones with no visible bacterial growth were measured with a ruler. The agar well diffusion assay was conducted with three biological replicates in technical triplicates (29).

#### 2.2.2 Broth microdilution assay for the determination of minimum inhibitory concentration (MIC) and minimum bactericidal concentration (MBC)

To prepare a dilution series in a 96 well plate, the well in the second column of the second row was filled with 50 μL tryptic soy broth (TSB:17 g/L casein peptone, 3 g/L soy peptone, 5 g/L NaCl, 2.5 g/L dipotassium phosphate, 2.5 g/L dextrose), 38 μL purified water, 2 μL dimethyl sulphoxide and 10 μL of cardamom essential oil. The remaining wells of the row were filled with 50 μL of a master mix that consisted of 50% (*v/v*) TSB, 48% (*v/v*) purified water and 2% (*v/v*) dimethyl sulphoxide. After mixing by pipetting up and down, 50 μL of the initially filled well in the second column were transferred to the adjacent well on the right, the suspension was mixed by pipetting and transferred to the next well until the serial dilution was complete. The pipette tips were discarded after every dilution. 50 μL from the last dilution were discarded. 50 μL of the 10^−2^ dilution, equal to approximately 5.0 × 10^5^ CFU/mL, were added to all wells that were previously filled. Thus, the final concentrations of the CEO were between 5.00% (*v/v*) and 0.01% (*v/v*). As a medium control, 100 μL TSB were added to one well (“MC”), and as a vehicle control 50 μL of the mastermix and 50 μL TSB were combined in another one (“VC –”). Another vehicle control consisted of 50 μL mastermix and 50 μL of bacterial suspension (“VC +”). A mixture of 25 μL TSB, 25 μL purified water and 50 μL bacterial suspension served as a negative control with undisturbed growth (“NC”). Also, a control with different dilutions of penicillin was included. For this, 25 μL TSB, 19 μL purified water, 1 μL dimethyl sulphoxide, and 5 μL of penicillin diluted in PW were combined in wells before 50 μL bacterial suspension from the 10^−2^ dilution were added. Penicillin dilutions with concentrations of 1 μg/mL, 10 μg/mL and 100 μg/mL were used as positive controls. The final concentrations of penicillin in a total volume of 100 μL were 0.05 μg/mL, 0.5 μg/mL and 5 μg/mL. The wells in row A and row H and in column 1 and column 12 were filled with purified water, the lid was placed on the microplate, and the edges of the lid were sealed with adhesive tape. Afterwards, the microplate was placed in the Tecan Infinite F200 Microplate Reader (Tecan Group AG, Männedorf, Switzerland). The microplate reader was set to 37°C and bacterial growth was recorded during the following 24 h. The kinetic cycle consisted of 48 cycles with 30 min kinetic intervals. Before the optical density was measured at 620 nm, the microplate was shaken linearly for 15 s at an amplitude of 3 mm. After 24 h, the MIC was determined by looking for the lowest concentration of cardamom essential oil that led to no increase of optical density. Then, 60 μL of all samples were transferred into petri dishes (60 mm diameter) and covered with warm, liquid TSB agar. After mixing the liquids by moving the petri dishes in the shape of a horizontal eight, the agar was left to dry in the bacterial safety cabinet before being incubated at 37°C for 48 h. MBC was determined by finding the lowest concentrations of cardamom essential oil that led to a mortality of 99.9%. These broth microdilution assays were performed with three biological replicates in triplicates.

#### 2.2.3 Crystal violet assay for the evaluation of biofilm formation

A 96 well microtiter plate was filled in the same manner with the same controls as described for the broth microdilution method. However, brain heart infusion [BHI, 27.5 g/L nutrient substrate, 2 g/L D-(+)-Glucose, 5 g/L NaCl, 2.5 g/L disodium hydrogen phosphate] broth with 0.2% (*w/v*) sucrose was used instead of TSB to enhance biofilm formation. For the serial dilution, a mastermix that was made of 50% (*v/v*) BHI with 0.2% (*w/v*) sucrose, 48% (*v/v*) purified water, and 2% (*v/v*) dimethyl sulphoxide was used. The microtiter plate was sealed with the microseal adhesive sealer by Bio-Rad Laboratories (Feldkirchen, Germany) and incubated at 37°C for 24 h without being shaken. The medium was carefully removed by pipetting without touching the bottoms of the wells, and 250 μL of PBS (0.14 M NaCl, 0.0027 M KCl, 0.01 M PO_4_^3−^+H_3_O^+^) were gently added into the wells and removed with a pipette. This washing step was repeated two more times. The 96 well plate was flipped onto a paper towel once and left to dry without a lid at 37°C for 10 min. Then, 150 μL of 0.1% (*w/v*) crystal violet diluted in purified water were added to the wells, and the microtiter plate was incubated for 15 min at RT. After removing the dye by pipetting, all wells were washed with 250 μL of PBS five times as described above, and the plate was left to dry for 10 min at 37°C without a lid. Afterwards, 150 μL 95% (*v/v)* ethanol diluted in PW were added to all wells and the microtiter plate was left in the orbital shaker incubator by Biosan (ES-20, Riga, Latvia) at RT and 250 rpm for 10 min. The 96 well microtiter plate was placed in the Tecan Infinite M200 Microplate Reader (Tecan Group AG, Männedorf, Switzerland). Before the measurement, the 96 well plate was shaken linearly for 15 s at an amplitude of 3 mm. The optical density was measured at 550 nm. To normalise the data, the average optical density value of the medium control was subtracted from all absorbance values. The reduction in biofilm formation was visualised by calculating the inhibitory rate using the following Equation 1:

$$\mathrm{inhibitory}\;\mathrm{rate}\;=\left(1-\frac{\mathrm{sample}}{\mathrm{negative}\;\mathrm{control}}\right)\;\times 100\text{\%}$$ (1)

The crystal violet assay was done with triplicates of three biological replicates (30).

#### 2.2.4 Methylthiazolyldiphenyl-tetrazolium bromide (MTT) assay for the evaluation of cell viability

A 96 well microtiter plate was filled in the same manner as described for the crystal violet assay with the same controls, but the number of samples with cardamom essential oil was reduced to 5.00%, 2.50%, 1.25%, 0.63%, and 0.31%. Afterwards, the microtiter plate was sealed with the microseal adhesive sealer by Bio-Rad Laboratories GmbH (Feldkirchen, Germany) and incubated at 37°C for 24 h without agitation. Then, 10 μL of MTT labeling reagent by Merck (Darmstadt, Germany), which consisted of 5 mg/mL MTT diluted in PBS, were added into the filled wells of the 96 well microtiter plate. The MTT was mixed with the medium in the wells by pipetting up and down approximately three times. Then, the microseal adhesive sealer and the lid were placed on the microtiter plate, and it was wrapped in aluminium foil. Afterwards, it was incubated at 37°C for 4 h before adding 100 μL dimethyl sulphoxide. The microtiter plate was left in the orbital shaker incubator at 37°C and 250 rpm for 15 min, and then placed into the Tecan Infinite M200 Microplate Reader where it was shaken linearly for 15 s at an amplitude of 3 mm before the optical densities OD_600_ and OD_700_ were measured at 600 nm and 700 nm, respectively. The OD_700_ values were measured as a reference wavelength, and after subtracting them from the OD_600_ values, the average (OD_600_—OD_700_) value of the medium control was subtracted from all previously calculated absorbance values to normalise them. This is described by the following Equation 2:

$$\mathrm{normalised}\;OD_{600}\;=\;(OD_{600}-OD_{700}{)}_{\mathrm{sample}}-(OD_{600}-OD_{700}{)}_{\mathrm{medium}\;\mathrm{control}\;\mathrm{average}}$$ (2)

The inhibitory rate of cell viability was calculated as described in Equation 1. Each experiment was performed in three biological replicates with three technical replicates each (31).

#### 2.2.5 Determination of virulence gene expression by quantitative kinetic PCR (qPCR)

*S. mutans* cells were grown in BHI medium with 0.2% (*w/v*) sucrose to promote biofilm formation. The bacterial suspension was diluted to a final optical density of 0.35 using Equation 3:

$$X_{\mathrm{ml}}\;=\;\frac{0.35\;\times 10\;\mathrm{mL}}{OD_{\mathrm{measured}}}$$ (3)

X_mL_ describes the volume of the bacterial suspension that is required to reach an optical density of 0.35 in a total volume of 10 mL. Thus, 10 mL—X_mL_ equals the amount of BHI with 0.2% (*w/v*) sucrose that is needed for the dilution, which was added to three autoclaved test tubes. After adding the calculated amount of bacterial suspension to each of the three test tubes, they were vortexed for one minute each. The bacterial suspension was dispensed into 24 microtubes, with treatments of penicillin and cardamom essential oil prepared accordingly. After 24 h incubation at 37°C and 250 rpm, cells were harvested, and RNA was extracted following the protocol by Rio et al., with modifications as follows (32): The initial centrifugation was at 12,000 × g and 4°C for 15 min. The final RNA pellet was washed with 1 mL of 70% (*v/v*) ethanol diluted in purified water at 1,400 rpm for 10 min, followed by centrifugation at 7,500 × g and 4°C for 5 min. After removing the supernatant, the pellets were air-dried at room temperature for 5 min and resuspended in 25 µL of nuclease-free water. RNA concentrations were measured using a NanoDrop™ OneC spectrophotometer (Thermo Fisher Scientific, Schwerte, Germany), then stored at −80°C.

Reverse transcription to complementary DNA (cDNA) and kinetic quantitative polymerase chain reaction (qPCR) were conducted as per Hornbacher et al., with minor modifications (33). Random nonamer primers were used for cDNA synthesis. qPCR was performed in 10 µL reaction mixture composed of 5 µL PowerTrackTM SYBR green mastermix (Thermo Fisher Scientific GmbH, Schwerte, Germany), 0.4 µL of each primer (Eurofins Genomics, Ebersberg, Germany), 2 µL of cDNA template and nuclease-free water. Seven target genes were analyzed, with primers listed in Table 1. Negative controls with nuclease-free water instead of cDNA templates were included. To quantify cDNA, standard curves were prepared by mixing 3.5 µL of the samples that were included as duplicates in one well and further 4-fold diluting this five times (see Supplementary Table S1 for final concentrations). qPCR was performed on a QuantStudio™ system (Thermo Fisher Scientific, Schwerte, Germany), using the gradient shown in Supplementary Table S2. Relative gene expression was calculated by dividing the absolute expression of six virulence genes by that of the housekeeping gene, *16S rRNA*. All negative controls lacked cDNA and showed no signal. The quality of the qPCR products was confirmed on a 2% (*w/v*) agarose gel, with no off-target products observed.

**Table 1:** Primer sequences used for quantitative kinetic PCR (qPCR) analysis [adopted from (30)].

Target Gene	Function/Role	Primer sequence (5′-3’)		Reference
		Forward primer	Reverse Primer	
*16S rRNA*	Housekeeping gene encoding 16S ribosomal RNA, used for normalization in qPCR studies of *S. mutans.*	CCTACGGGAGGCAGCAGTAG	CAACAGAGCTTTACGATCCGAAA	(30)
*ldh*	Lactate dehydrogenase; converts pyruvate to lactic acid, key for acidogenesis and enamel demineralization.	ACTTCACTTGATACTGCTCGTT	AACACCAGCTACATTGGCATGA	(56)
*gtfB*	Glucosyltransferase B; synthesizes mainly water-insoluble glucans, critical for sticky biofilm matrix and surface adhesion.	AGCAATGCAGCCAATCTACAAAT	ACGAACTTTGCCGTTATTGTCA	(13)
*gtfC*	Glucosyltransferase C; produces both soluble and insoluble glucans, contributes to structural stability of the biofilm matrix.	GTGCGCTACACCAATGACAGAG	GCCTACTGGAACCCAAACACCTA	(57)
*gtfD*	Glucosyltransferase D; synthesizes primarily soluble glucans, which serve as primers for GtfB-mediated insoluble glucan formation.	TGGCACCGCAATATGTCTCTTC	CAATCCGCAATAACCTGAATACCG	(57)
*gbpB*	Glucan-binding protein B; mediates adhesion to glucans and biofilm integrity.	ATGGCGGTTATGGACACGTT	TTTGGCCACCTTGAACACCT	(58)
*vicR*	Response regulator of the VicRK two-component system; regulates expression of *gtf* genes and other virulence determinants (biofilm, stress response).	TGACACGATTACAGCCTTTGATG	CGTCTAGTTCTGGTAACATTAAGTCCAATA	(59)

#### 2.2.6 Statistical analysis

All statistical analyses were performed using RStudio by Posit Software PBC (Boston, MA, USA). Data were tested for normality (Shapiro–Wilk) and variance homogeneity (Levene's test). Differences among groups were analyzed by Kruskal–Wallis followed by Dunn's *post hoc* test with adjusted *p* < 0.05. Results are expressed as mean ± SD.

## 3 Results

### 3.1 Antibacterial activity (agar well diffusion assay)

**Figure 1:**
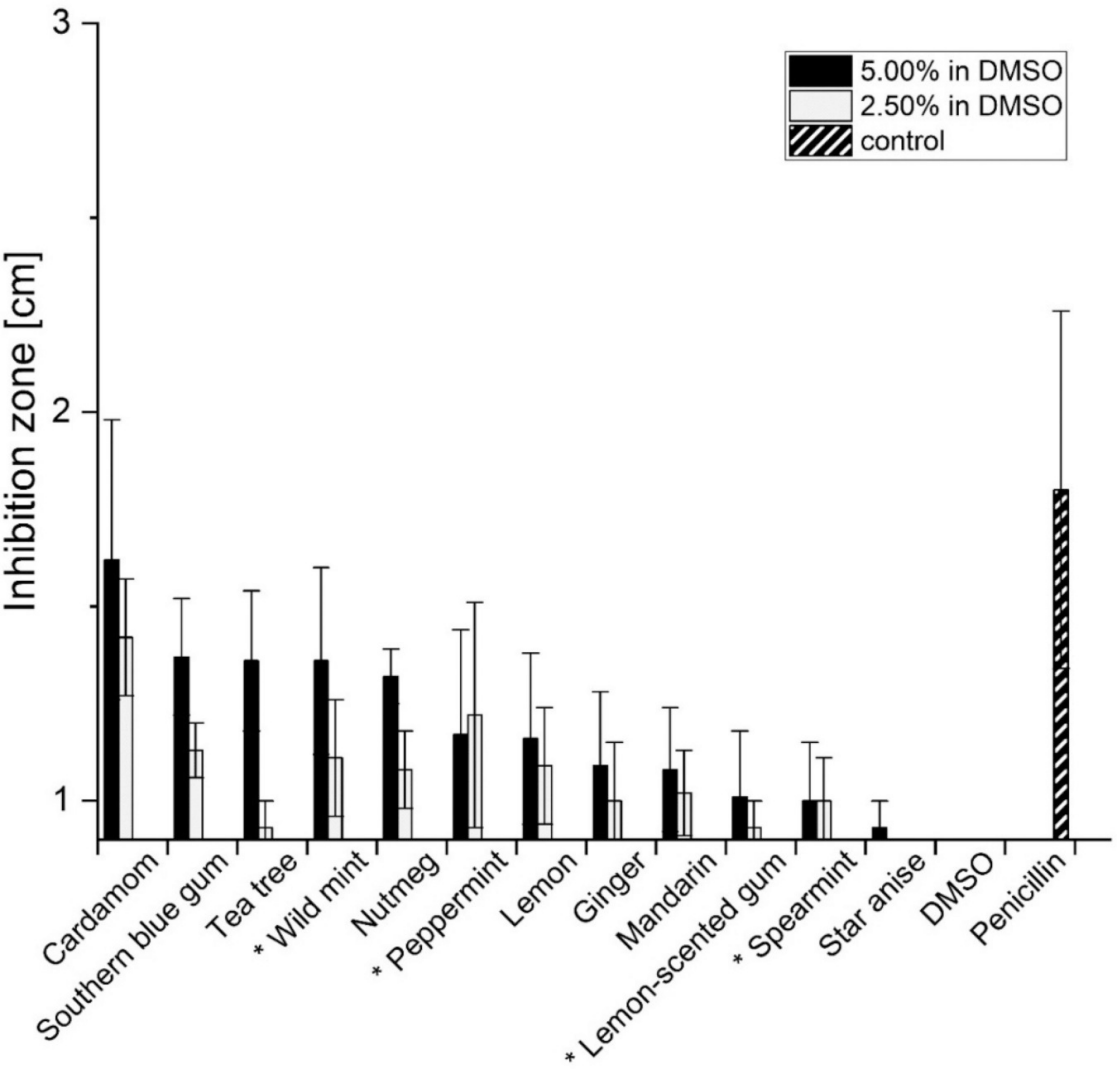
Inhibition zones (cm) of 12 essential oils against *S. mutans* compared with DMSO and 1 µg/mL penicillin (positive control). Black and grey bars represent essential oils at 5.00% and 2.50% (*v/v*) in DMSO, respectively. Values are mean ± SD (*n* = 9). Statistical significance relative to the DMSO control was determined by the Kruskal–Wallis test followed by Dunn's *post hoc* test (Bonferroni correction). Significant differences (*p* < 0.05) are indicated in the figure by asterisks.

Inhibition zones of the EOs had diameters between 10 mm and 15 mm on average, and essentially depended on EO concentration (Figure 1). Only peppermint EO and spearmint EO showed no decrease in inhibition zone diameters when being 40-fold diluted. Cardamom EO resulted in the largest inhibition zones that were significantly larger than those of lemon-scented gum EO, and star anise EO (Supplementary Table S3). Inhibition zones of southern blue gum EO, tea tree EO, wild mint EO and nutmeg EO were similar. Lemon, ginger, mandarin, lemon-scented gum and spearmint only caused very small inhibition zones, while star anise showed nearly no effect at all. All diluted EOs had a similar pH value between a pH of 6 and 7. The pH of the solvent DMSO was in the same range.

### 3.2 Minimum inhibitory concentration (MIC) and minimum bactericidal concentration (MBC)

**Figure 2:**
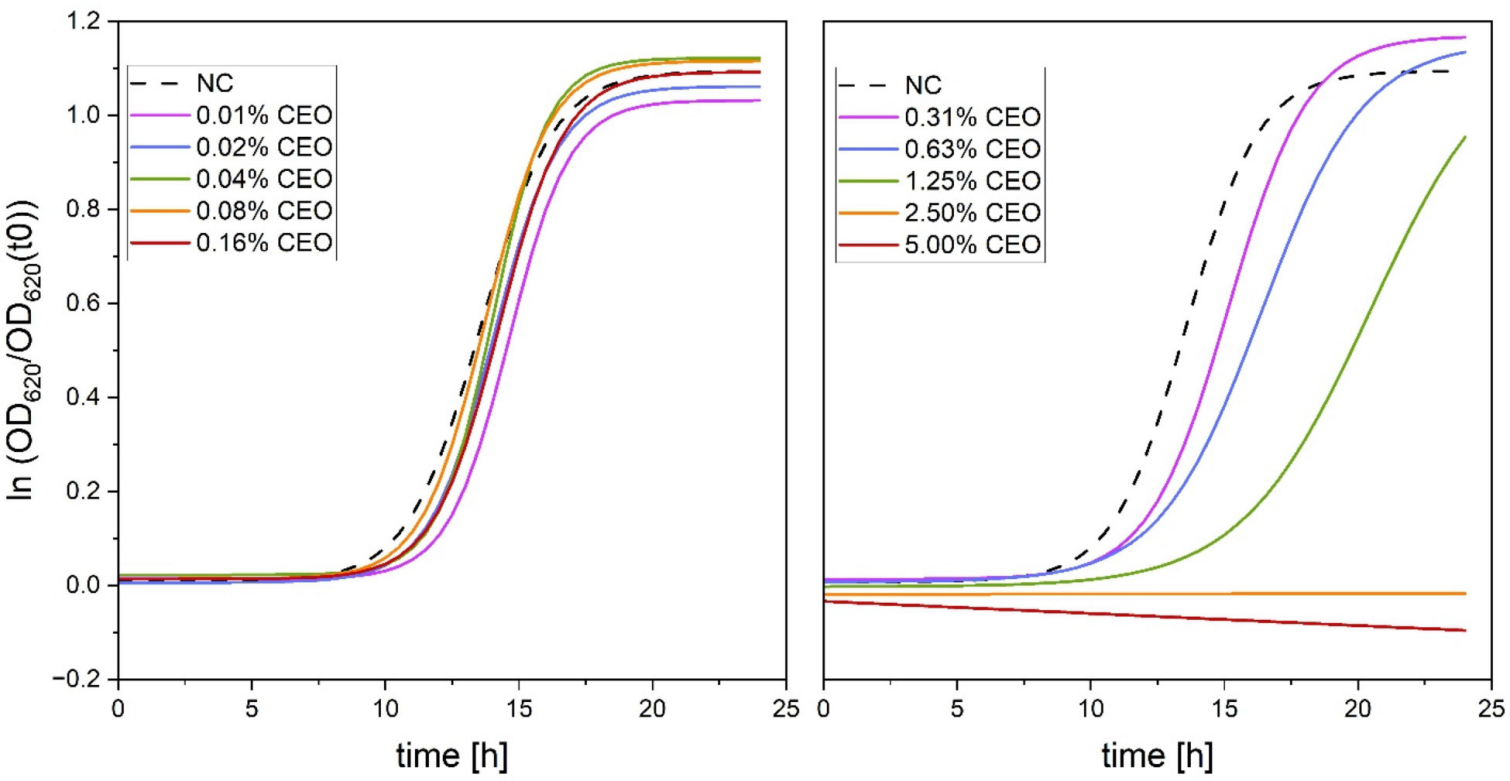
Impact of cardamom essential oil (CEO; 0.01–5.00%) on growth of *S. mutans*. The curves represent the mean normalized optical density [OD_620_/OD_620_(t₀)] from *n* = 9 replicates. NC indicates the untreated negative control. Growth at concentrations ≤0.31% CEO follows the control curve, whereas 0.63%–1.25% CEO show reduced slopes, and 2.50%-5.00% CEO completely inhibit bacterial growth.

The MIC of CEO against *S. mutans* was 2.50%, while the MBC was 5.00% (Figure 2). For the 2.50% CEO treatment and the 5.00% CEO treatment, no increase in optical density at 620 nm (OD_620_) was visible (Figure 2). The growth curves for the higher dilutions in a range between 0.01% and 0.16% CEO were not different from the reference growth curve. The growth curves for 0.31% and 0.63% CEO seemed to rise a little later. The growth curve for 1.25% CEO rose at a later time with a lower slope (Figure 2). Significant differences are shown in Supplementary Table S4.

### 3.3 Inhibition of biofilm formation by CEO

**Figure 3:**
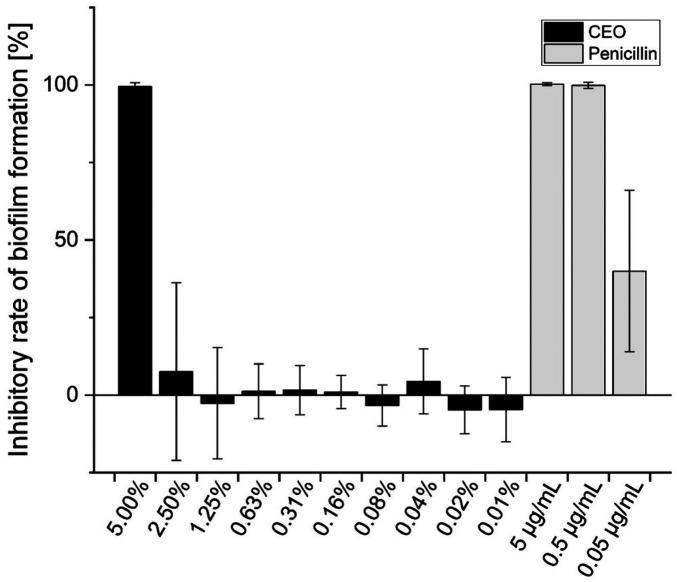
Inhibitory rate of *S. mutans* biofilm formation (%) in the presence of cardamom essential oil (CEO; 0.01%–5.00%) and penicillin (0.05–5 µg/mL). Dark bars represent CEO samples diluted in BHI broth with 0.2% (*w/v*) sucrose and 1% (*v/v*) DMSO; light bars correspond to penicillin controls prepared under the same conditions. Values are presented as mea*n* ± SD (*n* = 9). Statistical significance was determined by the Kruskal–Wallis test followed by Dunn's *post hoc* test with Bonferroni correction (*p* < 0.05).

As the broth microdilution assays demonstrated a decreased growth of *S. mutans* in the presence of CEO, the crystal violet assay was conducted to elucidate the influence on biofilm formation. At a concentration of 5.00% CEO, the biofilm formation decreased by 99.4 ± 1.25% (Figure 3). This decrease was significantly higher than that of the 0.01% CEO, 0.02% CEO, 0.08% CEO, and the VC+ samples (Supplementary Table S5). The inhibition of biofilm formation at 5.00% CEO was similar to those at 5 µg/mL and 0.5 µg/mL penicillin. A concentration of 2.50% CEO tended to marginally inhibit biofilm growth, even less than the 0.05 µg/mL penicillin control. All remaining samples with CEO concentrations between 0.01% and 1.25% displayed no significant inhibition (Figure 3).

### 3.4 Effect on cell viability

**Figure 4:**
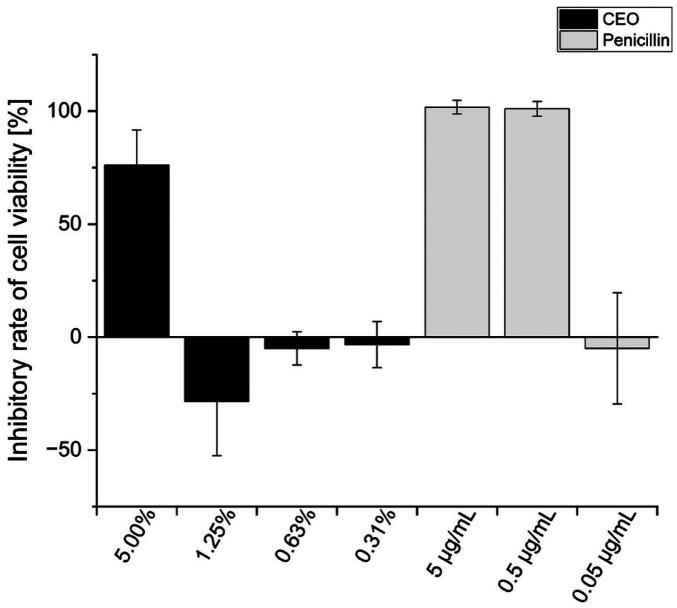
Impact of cardamom essential oil (CEO; 0.01%–5.00%) and penicillin (0.05–5 µg/mL) on cell viability of *S. mutans*. Dark bars represent CEO samples, and light bars represent penicillin controls, both diluted in brain heart infusion broth containing 0.2% (*w/v*) sucrose and 1% (*v/v*) DMSO. Values are mean ± SD (*n* = 9, except for penicillin 0.05 µg/mL, where *n* = 7 after outlier removal). According to the Kruskal–Wallis test followed by Dunn's *post hoc* test (Bonferroni correction), no statistically significant differences (*p* ≥ 0.05) were observed relative to the untreated control.

The MTT assay was conducted to analyse whether only the biofilm formation was disturbed or rather the viability of the organism. At a CEO concentration of 5.00%, the calculated cell viability of *S. mutans* decreased by 76.1 ± 15.51% (Figure 4). This was significantly different from the effects of 5 µg/mL or 0.5 µg/mL penicillin, and visibly below their inhibitory efficiency, as they led to inhibition rates of 101.7 ± 3.02% and 101.0 ± 3.28%, respectively (Supplementary Table S6). However, the inhibitory effect of 5.00% CEO was above that of 0.05 µg/mL penicillin. The CEO concentrations of 0.63% and 1.25% caused significantly lower inhibitions of *S. mutans* than the 5 µg/mL or 0.5 µg/mL penicillin controls. Although not significantly different, the 0.31% CEO samples also showed an inhibitory rate below 0%, thus indicating a lower effect on cell viability compared to the two penicillin controls mentioned above.

### 3.5 Effect on virulence gene expression

**Figure 5:**
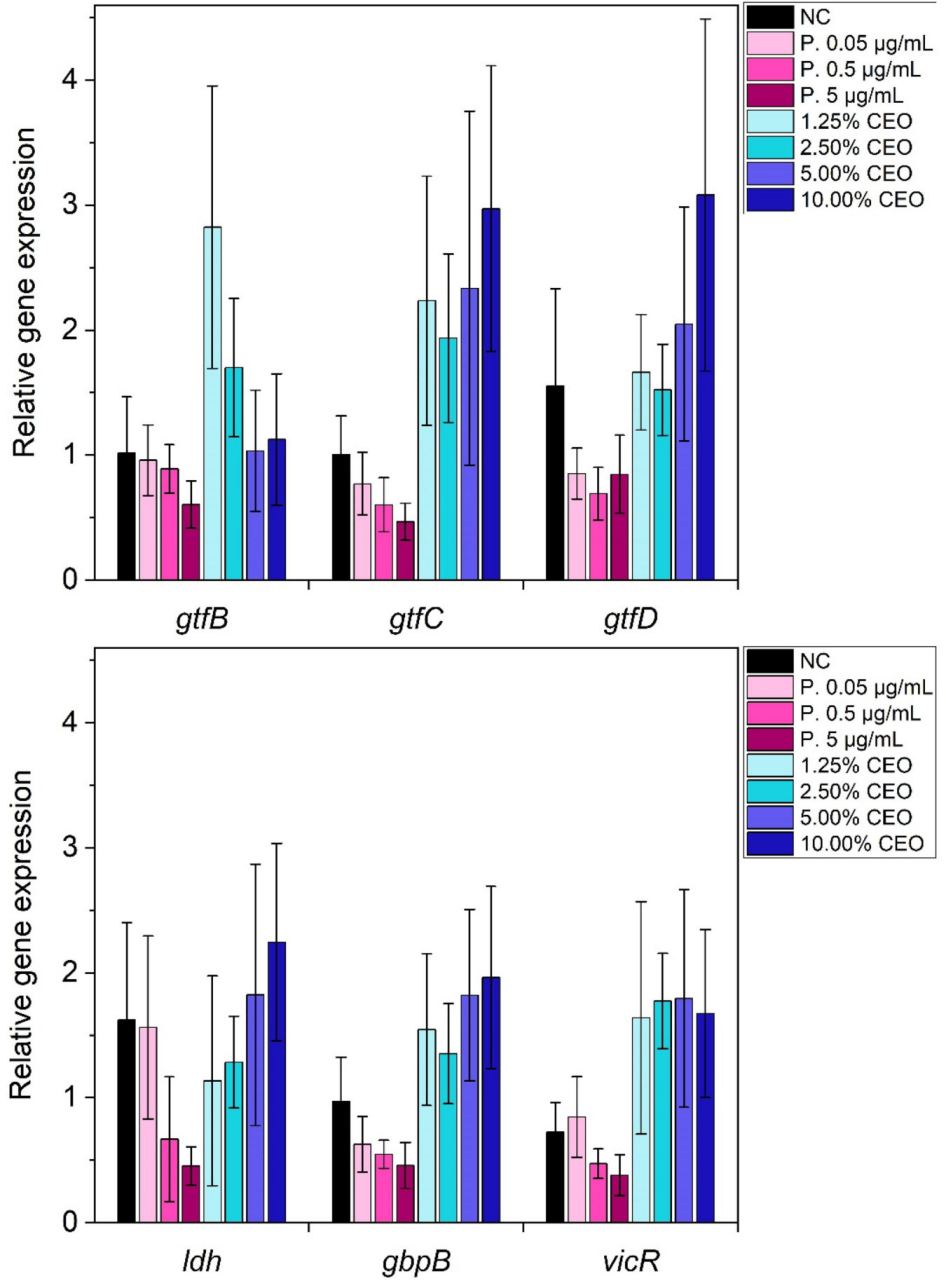
Impact of cardamom essential oil (CEO; 1.25–10.00%) and penicillin (0.05–5 µg/mL) on relative expression of virulence-associated genes (*gtfB, gtfC, gtfD, ldh, gbpB*, and *vicR*) in *S. mutans*. Expression levels were normalized to 16S rRNA and are presented as mean ± SD (*n* = 9). Statistical analysis was performed using the Kruskal–Wallis test followed by Dunn's *post hoc* test adjusted *p*-values (Bonferroni correction, *p* < 0.05).

qPCR was performed to elucidate the effects of CEO on the expression levels of six virulence genes. The *ldh* gene expression levels increased with an increase in the concentration of CEO (Figure 5). At 1.25% CEO and 2.50% CEO, the levels were below those of the untreated negative control (NC), while those of the 5.00% CEO and the 10.00% CEO treatments were above the NC. The relative *gtfB* gene expression levels of all CEO treatments were above those of the NC, and they gradually decreased with an increase in CEO concentration. The level of the 1.25% CEO treatment was nearly 3-fold higher than that of the NC. The 2.50% CEO treatment was approximately 1.5-fold higher than the NC, but the 5.00% CEO and the 10.00% CEO treatments were similar to the NC. As for the relative gene expressions of *gtfC*, *gtfD* and *gbpB* a similar trend was observed. From 1.25% CEO to 2.50% CEO, the level decreased slightly before increasing gradually to 5.00% CEO and 10.00% CEO. The relative expression levels at 10.00% CEO of *gtfC* and *gtfD* were higher than those of *gbpB*. In the case of *vicR* the relative gene expression levels of all CEO treatments were above those of the NC. The levels slightly increased from the 1.25% CEO treatment to the 2.50% CEO treatment and to the 5.00% CEO treatment. However, the level of the 10.00% CEO treatment was similar to that of the 1.25% CEO treatment. Overall, all four levels were not much different from each other but more than twice as high as the level of the NC. Significant differences are shown in Supplementary Table S7.

## 4 Discussion

This study demonstrated that cardamom essential oil (CEO) exhibits inhibitory effects against *S. mutans*, including growth suppression and biofilm inhibition *in vitro*. Among the twelve food-derived essential oils screened, CEO showed the strongest antibacterial activity, supporting its potential as a natural antimicrobial agent. The observed variability in the activity of different essential oils primarily depends on the plant species and their secondary metabolite composition. Factors such as plant origin, extraction method, storage conditions, and environmental parameters including temperature and humidity, further modulate this composition and thereby explain the variability in biological effects observed even among essential oils derived from the same plant species (24, 25, 29, 34–45). In accordance with this, the minimum inhibitory concentration (MIC) values reported in the literature for CEO and its derivatives also show significant variations. For example, while Karimi et al. (25) reported an MIC value of 0.03 mg/mL for CEO, Binimeliz et al. (24) reported a significantly higher value of 7.34 mg/mL for ethanolic extracts. These differences are considered to be related to variations in the extraction methods used and plant-derived variations, as well as differences in the concentration of 1,8-cineole, the primary bioactive component of CEO.

Compared to EOs that have been studied more extensively, such as clove or tea tree oil, the effects of CEO against cariogenic bacteria have been investigated in a relatively limited number of studies. The role of 1,8-cineole in CEO's antimicrobial activity is also supported by studies carried out by Yang et al. (46). In the mentioned study, it was reported that the minimal microbicidal concentrations (MMC) of *Litsea cubeba* essential oil (LC-EO) against cariogenic bacteria ranged from 0.375 to 1.5 mg/mL for *S. mutans* (without biofilm), and that biofilm-associated MMCs were approximately double that value. Interestingly, the 1,8-cineole levels estimated in the present CEO samples exceeded those MMCs, suggesting that other constituents within the oil may modulate its efficacy or that bacterial aggregation within biofilms confers partial protection.

CEO typically contains 20%–30% 1,8-cineole, along with *α*-terpineol, linalool, and sabinene (46–49). These compounds are known to disrupt bacterial membranes and enzymatic activity, yet their specific interactions remain unclear. The current data suggest that while 1,8-cineole represents the principal antimicrobial component, its activity may be attenuated by synergistic or antagonistic effects of coexisting compounds.

Growth curve analyses indicate that CEO exhibits a concentration-dependent inhibition profile. While limited effects were observed at low concentrations, partial inhibition was observed at moderate concentrations, and significant growth suppression was observed at high concentrations. This indicates that even concentrations below the MIC can disrupt bacterial physiology and that sublethal effects should not be disregarded. Regarding biofilm inhibition, this study presents the first direct evidence of CEO activity against *S. mutans* biofilms. Compared to other EOs such as clove or lemon, the fact that CEO requires higher concentrations to achieve similar biofilm suppression suggests that it may have lower potential in this context (44, 50, 51). Nevertheless, 5% CEO disrupted biofilms to a degree comparable with 5 µg/mL penicillin, though approximately 24% of cells remained viable. These findings indicate that CEO interferes with biofilm integrity rather than fully eradicating planktonic cells, consistent with the hypothesis proposed by Vazquez et al. (52), who suggested that 1,8-cineole can penetrate biofilms and create micropores that destabilize the structure without complete cell death.

He et al. demonstrated that cinnamaldehyde exhibited a higher bactericidal effect against *S. mutans* than CEO (30). This indicates that the antimicrobial activity of essential oils depends not only on their composition but also on the concentration of their active components. Indeed, Song et al. (42) demonstrated that essential oils such as tea tree oil must be applied at high concentrations to completely eliminate *S. mutans*. Similar to these studies, the present study also found that the antibacterial effect of CEO is concentration-dependent and that a significant decrease in cell viability was observed only at high concentrations.

The observed upregulation of virulence-associated genes under cardamom essential oil exposure may represent a bacterial stress response. Although the qPCR assays included CEO concentrations up to bactericidal levels, these experiments were performed under biofilm-forming conditions, where spatial heterogeneity in cell viability is expected. As reported for *S. mutans* and other biofilm-forming species, bactericidal agents may effectively kill cells on the biofilm surface while subpopulations within deeper layers remain viable and metabolically active. Therefore, the detected transcripts likely originate from surviving cells within the biofilm rather than from lysed ones. This approach was intended to approximate oral biofilms, where complete eradication rarely occurs and stress-induced gene regulation contributes to persistence and adaptation. For this reason, it is thought that *S. mutans* increases the expression of adhesion- and biofilm-related factors to maintain colonization capacity under suboptimal conditions caused by antibacterial compounds.

The effects of CEO on virulence gene expression are similar to those of ficin but opposite to those of cinnamaldehyde. While cinnamaldehyde reduces *gtfB* expression, ficin and CEO (especially at concentrations of 1.25% and 2.50%) significantly increase it. Similar trends were observed for *gtfC* and *gtfD*, and it is thought that these increases may support the production of water soluble and insoluble glucan (19, 30).

Such transcriptional changes may be associated with *S. mutans* developing a compensatory response under adverse conditions, such as biofilm formation inhibition or cell damage. The increased expression of *gbpB*, which is associated with surface adhesion, and *vicR*, which regulates multiple virulence genes, under the influence of CEO and ficin also supports this interpretation. It is observed that the increase in *vicR* expression is consistent with the upregulation of the *gbpB*, *gtfB*, *gtfC*, and *gtfD* genes (19, 30).

In comparison, *ldh* gene expression is not regulated by *vicR* and exhibits a concentration-dependent response to CEO. The decrease observed at low concentrations is similar to the reported effects of cinnamaldehyde on *ldh* expression, while the increase observed at high concentrations is similar to the response described for ficin (19). Given that the MIC of CEO is 5.00%, it is thought that the increase *ldh* expression observed at inhibitory concentrations may be related to an increased energy requirement due to membrane disruption and, accordingly, may enhance the conversion of pyruvate to lactate., which could increase cellular energy demand thereby enhance pyruvate conversion to lactate.

Although CEO was found to exhibit significant inhibitory effects on *S. mutans*, the specific mechanism underlying this activity could not be directly demostrated in this study. On the basis of the observed phenotypic and transcriptional changes, it is thought that CEO may interfere with bacterial membrane integrity, biofilm-related functions, or stress response pathways. However, direct mechanistic analyses, such as membrane integrity assays, electron microscopy, or membrane potential measurements, are required to validate these hypotheses. In addition, the safety profile of CEO in mammalian cells has not yet been evaluated. Cytotoxicity and hemolytic tests are required to determine its potential applicability in clinical settings.

## 5 Conclusions

This study provides significant findings that offer a deeper insight into the antibacterial and antibiofilm effects of cardamom essential oil (CEO) against *S. mutans*. Notably, a concentration of 5.00% CEO was identified as bactericidal and strongly inhibited biofilm formation. The correlation between gene expression analyses and phenotypic observations indicates that CEO has a biologically significant effect on *S. mutans*. These findings suggest that it may have the potential to prevent *S. mutans* colonization on tooth surfaces and hold promise for the development of natural oral care products.

## 6 Limitations and future perspectives

In this study, the use of 16S rRNA as a reference gene is a limitation. Although it has been frequently applied in previous *S. mutans* studies e.g., (30), several reports caution that its expression can vary under stress conditions, which may affect normalization accuracy. Therefore we cannot fully exclude that normalization to 16S rRNA contributed to the magnitude of some changes However, the consistent direction observed across multiple virulence genes and the consistency with phenotypic data support the conclusion that the results reflect a biological stress response. In future studies, it is recommended that alternative reference genes for *S. mutans* (e.g., *gyrA, recA, tuf, rpoB, atpD*) be validated using methods such as geNorm, NormFinder, or BestKeeper, (53–55).

In addition, this study was conducted using mono-species cultures of *S. mutans*. However, dental caries is a polymicrobial disease, and the inhibitory effects of CEO observed here may not fully translate to mixed-species biofilms or *in vivo* conditions. Future studies should therefore include additional cariogenic species and polymicrobial biofilm models to better reflect the clinical situation.

Another limitation of this study is the lack of direct evidence explaining the mechanism by which the CEO inhibits *S. mutans*. Although the results indicate possible effects on processes related to bacterial physiology and virulence, further studies involving targeted mechanistic approaches are needed. Additionally, the use of 16S rRNA as a reference gene may constitute a limitation, and future studies should consider alternative reference genes such as *gyrA*. An additional limitation of this study is that cytotoxicity and biocompatibility assessments of the CEO were not performed in mammalian cell lines. Future studies should include methods such as cytotoxicity testing and hemolysis testing in epithelial cell lines and erythrocytes, respectively to evaluate safety. It is recommended that future research should focus on evaluating CEO activity in polymicrobial and *in vivo* models to better reflect clinical conditions, as well as on improving its aqueous solubility and cost-effective formulation for practical application in oral hygiene products.

## Data Availability

The original contributions presented in the study are included in the article/Supplementary Material, further inquiries can be directed to the corresponding author/s.
