# Supplementary material for: In vitro inhibition of Streptococcus mutans by cardamom essential oil
Source: Front Oral Health. 2026 May 11;7:1796712. doi: 10.3389/froh.2026.1796712 (PMC13199289; doi:10.3389/froh.2026.1796712)
Supplement: Supplementary file 1 [file Datasheet1.docx]

**In Vitro Inhibition of *Streptococcus mutans* by Cardamom Essential Oil**

Nanki J. Singh^1,2^, Gizem Kezer^1^, Marcus A. Horn^2^, Silke Hillebrand^3^, Tuba Esatbeyoglu^1*^

^1^Department of Molecular Food Chemistry and Food Development, Institute of Food and One Health, Gottfried Wilhelm Leibniz University Hannover, Am Kleinen Felde 30, 30167 Hannover, Germany

^2^Institute of Microbiology, Gottfried Wilhelm Leibniz University Hannover, Herrenhäuser Straße 2, 30419 Hannover, Germany

^3^Symrise AG, Muehlenfeldstraße 1, 37603 Holzminden, Germany

***Corresponding author:**

Prof. Dr. Tuba Esatbeyoglu

Institute of Food and One Health

Gottfried Wilhelm Leibniz University Hannover

Am Kleinen Felde 30

30167 Hannover, Germany

Tel: +49 511 762 5589

Email: esatbeyoglu@foh.uni-hannover.de

**Supplementary Figure Captions**

**Supplementary Figure 1.** Structural formulas of bioactive compounds in cardamom essential oil. Cardamom essential oil consists of compounds that are capable of inhibiting bacteria, such as 1,8-cineole, α-terpineol, linalool, linalyl acetate, D-limonene and sabinene.


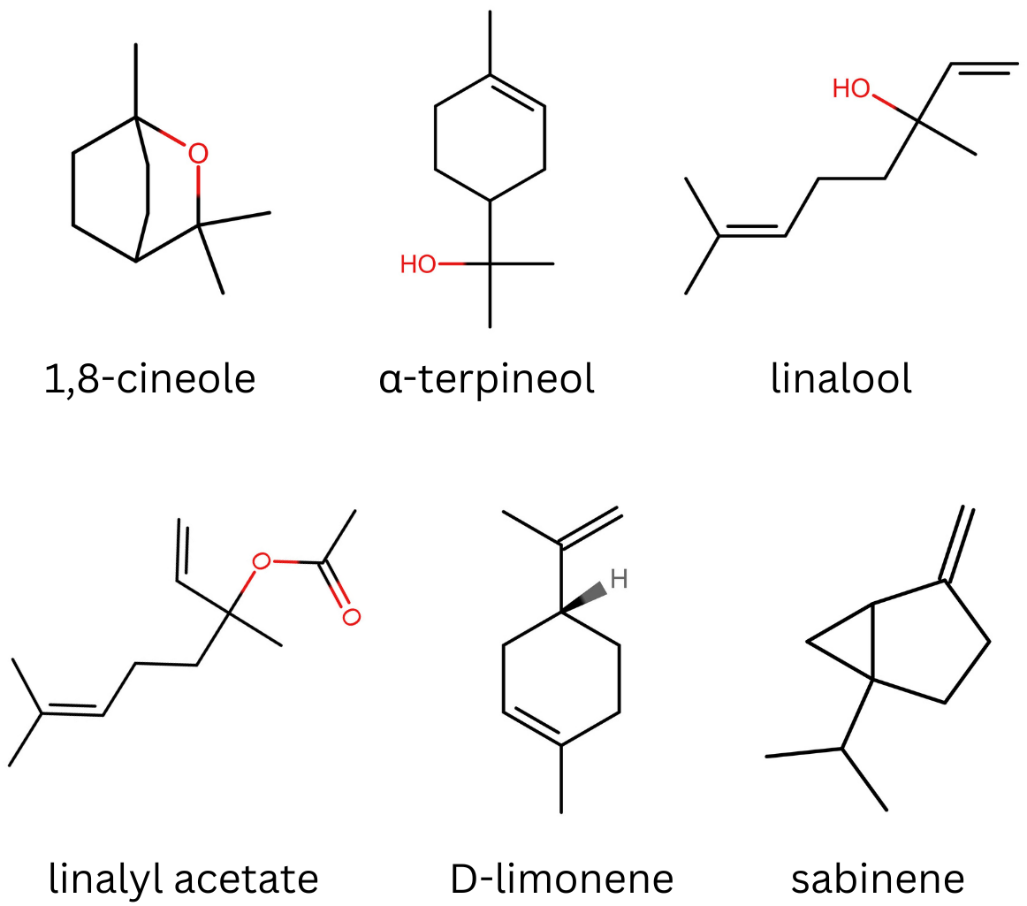


**Supplementary Tables**

**Supplementary Table 1.** Standards for qPCR.

| **Standard** | **Dilution** | **Concentration** |
| --- | --- | --- |
| 1 | 1 | 1.000 |
| 2 | 1:4 | 0.250 |
| 3 | 1:16 | 0.063 |
| 4 | 1:64 | 0.016 |
| 5 | 1:256 | 0.004 |
| 6 | 1:1024 | 0.001 |

**Supplementary Table 2.** Gradient for qPCR.

| **Stage** | **Temperature [°C]** | **Time [min:s]** | **Number of cycles** |
| --- | --- | --- | --- |
| **Hold stage** | 50 | 02:00 |  |
|  | 95 | 10:00 |  |
| **PCR stage** | 95 | 00:15 | 40 x |
|  | 60 | 01:00 |  |
| **Melt curve stage** | 95 | 00:15 |  |
|  | 60 | 01:00 |  |
|  | 95 | 00:01 |  |

**Supplementary Table 3.** Significant differences during the agar well diffusion assay with *p*-values < 0.05.

**a) *p* -values < 0.05 among the 5.00% EOs diluted in DMSO.**

|  | **Car** | **SbG** | **TeT** | **WiM** | **Nut** | **Pen** |
| --- | --- | --- | --- | --- | --- | --- |
| **LsG** | 2.52e-02 | n.s. | n.s. | n.s. | n.s. | n.s. |
| **StA** | 1.79e-03 | 3.39e-02 | 4.55e-02 | 8.70e-02 | n.s. | n.s. |
| **Spe** | 2.65e-02 | n.s. | n.s. | n.s. | n.s. | n.s. |
| **DMSO** | 3.39e-04 | 8.16e-03 | 1.12e-02 | 2.28e-02 | 1.46e-02 | 4.00e-02 |

Abbreviations: LsG = Lemon-scented gum; StA = Star anise; Spe = Spearmint; Car = Cardamom; SbG = Southern blue gum; TeT = Tea tree; WiM = Wild mint; Nut = Nutmeg; Pen = Penicillin; n.s. = no significant difference. The table summarises all p-values of samples with a significant difference according to Dunn's test. The p-values were adjusted with the Bonferroni method.

**b) *p*-values < 0.05 among the 2.50% EOs diluted in DMSO.**

|  | **LsG** | **StA** | **TeT** | **DMSO** |
| --- | --- | --- | --- | --- |
| **Car** | 1.88e-03 | 1.48e-04 | 1.88e-03 | 1.48e-04 |
| **Pen** | n.s. | 2.30e-02 | n.s. | 2.30e-02 |

Abbreviations: LsG = Lemon-scented gum; StA = Star anise; TeT = Tea tree; Car = Cardamom; Pen = Penicillin; n.s. = no significant difference. The table summarises all p-values of samples with a significant difference according to Dunn's test. The p-values were adjusted with the Bonferroni method.

**Supplementary Table 4.** Significant differences of the broth microdilution assay with *p*-values < 0.05.

|  | **0.01% CEO** | **0.02% CEO** | **0.04% CEO** | **0.08% CEO** | **0.16% CEO** | **0.31% CEO** | **0.63% CEO** | **VC +** | **NC** |
| --- | --- | --- | --- | --- | --- | --- | --- | --- | --- |
| **2.50% CEO** | n.s. | n.s. | 4.66e-02 | n.s. | n.s. | n.s. | n.s. | 3.04e-03 | n.s. |
| **5.00% CEO** | n.s. | n.s. | 3.09e-02 | n.s. | n.s. | 3.40e-02 | n.s. | 1.89e-03 | 3.51e-02 |
| **MC** | n.s. | n.s. | n.s. | n.s. | n.s. | n.s. | n.s. | 3.39e-03 | n.s. |
| **VC –** | 3.59e-02 | 1.99e-02 | 4.57e-03 | 1.49e-02 | 1.56e-02 | 5.08e-03 | 1.43e-02 | 2.08e-04 | 5.26e-03 |
| **Pen. 5 μg/mL** | n.s. | n.s. | n.s. | n.s. | n.s. | n.s. | n.s. | 5.97e-03 | n.s. |
| **Pen. 0.5 μg/mL** | n.s. | n.s. | 8.47e-03 | 2.66e-02 | 2.78e-02 | 9.39e-03 | 2.55e-02 | 4.24e-04 | 9.72e-03 |
| **Pen. 0.05 μg/mL** | 1.84e-02 | 9.94e-03 | 2.16e-03 | 7.38e-03 | 7.73e-03 | 2.41e-03 | 7.05e-03 | 8.87e-05 | 2.50e-03 |

Abbreviations: CEO = Cardamom essential oil; VC + = vehicle control with 50 μL mastermix and 50 μL of bacterial suspension; NC = negative control with 25 μL tryptic soy broth, 25 μL purified water and 50 μL bacterial suspension; MC = medium control with 100 μL tryptic soy broth; VC – = vehicle control with 50 μL mastermix and 50 μL tryptic soy broth; Pen = Penicillin; n.s. = no significant difference. The table summarises all p-values of samples with a significant difference according to Dunn's test. The p-values were adjusted with the Bonferroni method.

**Supplementary Table 5.** Significant differences during the crystal violet assay with *p*-values < 0.05.

|  | **0.01% CEO** | **0.02% CEO** | **0.08% CEO** | **0.16% CEO** | **0.31% CEO** | **0.63% CEO** | **1.25% CEO** | **VC +** | **NC** |
| --- | --- | --- | --- | --- | --- | --- | --- | --- | --- |
| **5.00% CEO** | 9.11e-03 | 3.81e-03 | 1.16e-02 | n.s. | n.s. | n.s. | n.s. | 2.37e-03 | n.s. |
| **MC** | 8.91e-04 | 3.37e-04 | 1.17e-03 | 1.84e-02 | 3.06e-02 | 1.51e-02 | 1.96e-02 | 1.99e-04 | 1.40e-02 |
| **VC –** | 2.37e-03 | 9.35e-04 | 3.08e-03 | 4.27e-02 | n.s. | 3.54e-02 | 4.53e-02 | 5.64e-04 | 3.30e-02 |
| **Pen. 5 μg/mL** | 7.75e-04 | 2.91e-04 | 1.02e-04 | 1.63e-02 | 2.73e-02 | 1.13e-02 | 1.74e-02 | 1.71e-04 | 1.24e-02 |
| **Pen. 0.5 μg/mL** | 2.67e-03 | 1.06e-03 | 3.47e-03 | 4.72e-02 | n.s. | 3.93e-02 | n.s. | 6.41e-04 | 3.66e-02 |

Abbreviations: CEO = Cardamom essential oil; VC + = vehicle control with 50 μL mastermix and 50 μL of bacterial suspension; NC = negative control with 25 μLbrain heart infusion broth with 0.2% (w/v) sucrose, 25 μL purified water and 50 μL bacterial suspension; MC = medium control with 100 μL tryptic soy broth; VC – = vehicle control with 50 μL mastermix and 50 μL tryptic soy broth; Pen = penicillin; n.s. = no significant difference. The table summarises all p-values of samples with a significant difference according to Dunn's test. The *p*-values were adjusted with the Bonferroni method.

**Supplementary Table 6.** Significant differences of treatments during the MTT assay with *p*-values < 0.05.

|  | **0.31% CEO** | **0.63% CEO** | **1.25% CEO** | **2.50% CEO** | **Pen. 0.05 μg/mL** | **VC +** | **NC** |
| --- | --- | --- | --- | --- | --- | --- | --- |
| **5.00% CEO** | n.s. | n.s. | n.s. | 2.16e-03 | n.s. | n.s. | n.s. |
| **MC** | n.s. | n.s. | 9.15e-04 | 5.67e-06 | 2.63e-02 | n.s. | n.s. |
| **VC –** | 1.68e-02 | 2.94e-03 | 2.75e-05 | 8.48e-08 | 1.38e-03 | 1.11e-02 | 2.22e-02 |
| **Pen. 5 μg/mL** | n.s. | 1.27e-02 | 1.66e-04 | 7.21e-07 | 6.30e-03 | 4.30e-02 | n.s. |
| **Pen. 0.5 μg/mL** | n.s. | 2.22e-02 | 3.31e-04 | 1.66e-06 | 1.13e-02 | n.s. | n.s. |

Abbreviations: CEO = Cardamom essential oil; VC + = vehicle control with 50 μL mastermix and 50 μL of bacterial suspension; NC = negative control with 25 μL brain heart infusion broth with 0.2% (w/v) sucrose, 25 μL purified water and 50 μL bacterial suspension; MC = medium control with 100 μL tryptic soy broth; VC – = vehicle control with 50 μL mastermix and 50 μL tryptic soy broth; Pen = penicillin; n.s. = no significant difference. The table summarises all p-values of samples with a significant difference according to Dunn's test. The *p*-values were adjusted with the Bonferroni method.

**Supplementary Table 7.** Significant differences of treatments during the qPCR with *p*-values < 0.05.

**a) *16S rRNA* gene expression**

|  | **1-RT** | **4-RT** | **5-RT** | **2.5% CEO** | **5% CEO** | **10% CEO** | **no temp.** |
| --- | --- | --- | --- | --- | --- | --- | --- |
| **Pen. 0.05 μg/mL** | 1.38E-03 | 7.91E-04 | 7.91E-04 | 3.28E-02 | 4.32E-04 | 1.38E-04 | 7.91E-04 |
| **Pen. 0.5 μg/mL** | 3.12E-02 | 1.96E-02 | 1.96E-02 | n.s. | 3.92E-02 | 1.59E-02 | 1.96E-02 |
| **Pen. 5 μg/mL** | n.s. | 4.68E-02 | 4.68E-02 | n.s. | n.s. | n.s. | 4.68E-02 |

**b) *ldh* gene expression**

|  | **1-RT** | **4-RT** | **5-RT** | **2.5% CEO** | **5% CEO** | **10% CEO** | **no temp.** |
| --- | --- | --- | --- | --- | --- | --- | --- |
| **NC** | 4.39E-02 | 3.50E-02 | 4.39E-02 | n.s. | n.s. | n.s. | 1.88E-02 |
| **Pen. 0.05 μg/mL** | 1.00E-03 | 7.56E-04 | 1.00E-03 | 3.44E-02 | 2.32E-03 | 7.44E-03 | 3.51E-04 |

**c) *gtfB* gene expression**

|  | **1-RT** | **4-RT** | **5-RT** | **5% CEO** | **10% CEO** | **no temp.** |
| --- | --- | --- | --- | --- | --- | --- |
| **Pen. 0.05 μg/mL** | 2.98E-03 | 2.85E-03 | 1.16E-03 | 1.03E-04 | 1.09E-04 | 2.16E-04 |
| **Pen. 0.5 μg/mL** | n.s. | n.s. | n.s. | 4.12E-02 | 4.27E-02 | 1.56E-02 |
| **1.25% CEO** | n.s. | n.s. | n.s. | n.s. | n.s. | 2.65E-02 |

**d) *gtfC* gene expression**

|  | **1-RT** | **4-RT** | **5-RT** | **5% CEO** | **10% CEO** | **no temp.** |
| --- | --- | --- | --- | --- | --- | --- |
| **Pen. 0.05 μg/mL** | 1.37E-03 | 5.63E-03 | 1.19E-02 | 2.76E-03 | 1.99E-02 | 3.31E-04 |

**e) *gtfD* gene expression**

|  | **1-RT** | **4-RT** | **5-RT** | **2.5% CEO** | **5% CEO** | **10% CEO** | **no temp.** |
| --- | --- | --- | --- | --- | --- | --- | --- |
| **Pen. 0.05 μg/mL** | 1.43E-03 | 3.81E-03 | 2.24E-03 | 2.57E-02 | 8.26E-04 | 1.31E-02 | 5.60E-04 |

**f) *gbpB* gene expression**

|  | **1-RT** | **4-RT** | **5-RT** | **2.5% CEO** | **5% CEO** | **10% CEO** | **no temp.** |
| --- | --- | --- | --- | --- | --- | --- | --- |
| **NC** | n.s. | n.s. | n.s. | n.s. | n.s. | n.s. | 4.73E-02 |
| **Pen. 0.05 μg/mL** | 9.54E-04 | 1.26E-03 | 7.20E-04 | 4.49E-02 | 8.24E-04 | 2.59E-04 | 3.68E-04 |
| **Pen. 0.5 μg/mL** | n.s. | n.s. | n.s. | n.s. | n.s. | n.s. | 4.67E-02 |

**g) *vicR* gene expression**

|  | **1-RT** | **4-RT** | **5-RT** | **5% CEO** | **10% CEO** | **no temp.** | **Pen. 5 μg/mL** |
| --- | --- | --- | --- | --- | --- | --- | --- |
| **Pen. 0.05 μg/mL** | 6.35E-04 | 6.99E-04 | 1.10E-02 | 3.85E-04 | 1.46E-04 | 1.29E-04 | 3.23E-02 |

Abbreviations: -RT = samples that were not treated with reverse transcriptase during cDNA synthesis; CEO = Cardamom essential oil; no temp. = control with nuclease-free water instead of cDNA; Pen = penicillin; NC = negative control; n.s. = no significant difference. The table summarises all *p*-values of samples with a significant difference according to Dunn's test. The *p*-values were adjusted with the Bonferroni method.
